# Supplementary material for: Late Dialysis Modality Education Could Negatively Predict Peritoneal Dialysis Selection
Source: J Clin Med. 2022 Jul 13;11(14):4042. doi: 10.3390/jcm11144042 (PMC9315828; doi:10.3390/jcm11144042)
Supplement: Supplementary file 1 [file jcm-11-04042-s001.zip › jcm-1740957-supplementary.pdf]

Supplementary materials

Number of Supplemental Table: 2 Tables

Number of Supplemental Figure: 2 Figures

Table S1. Healthcare professionals primarily responsible for RRT education

	All patients	Groups with HD	Groups with PD	P-value
Nurse specialists	221	180 (81.4%)	41 (18.6%)	< 0.05
Nephrologists	134	122 (91.0%)	12 (9.0%)	

Abbreviations: RRT, renal replacement therapy; HD, hemodialysis; PD, peritoneal dialysis.

Table S2. Results of univariate logistic regression analyses associated with PD selection

Variables	OR (95%CI)	P-value
Age (per 10 years)	0.63 (0.51–0.78)	<0.01
Sex (Female)	0.69 (0.35–1.37)	0.29
Welfare public assistance	0.47 (0.06–3.65)	0.47
Living alone	0.70 (0.32–1.57)	0.39
Charlson comorbidity index (per 1)	0.72 (0.60–0.88)	<0.01
Geriatric nutritional risk index (per 10)	1.41 (1.11–1.80)	<0.01
eGFR at first visit to the nephrology department (per 1 ml/min/1.73 m^2)	1.01 (0.99–1.02)	0.49
eGFR at RRT education (per 1 ml/min/1.73 m^2)	1.16 (1.05–1.28)	< 0.01
eGFR decline rate for 6 months before dialysis initiation (%)	0.95 (0.81–1.10)	0.46
eGFR at dialysis initiation (per 1 ml/min/1.73 m^2)	1.04 (0.90–1.20)	0.60

Abbreviations: PD, peritoneal dialysis; OR, odds ratio; CI, confidence interval; eGFR, estimated glomerular filtration rate; RRT, renal replacement therapy.

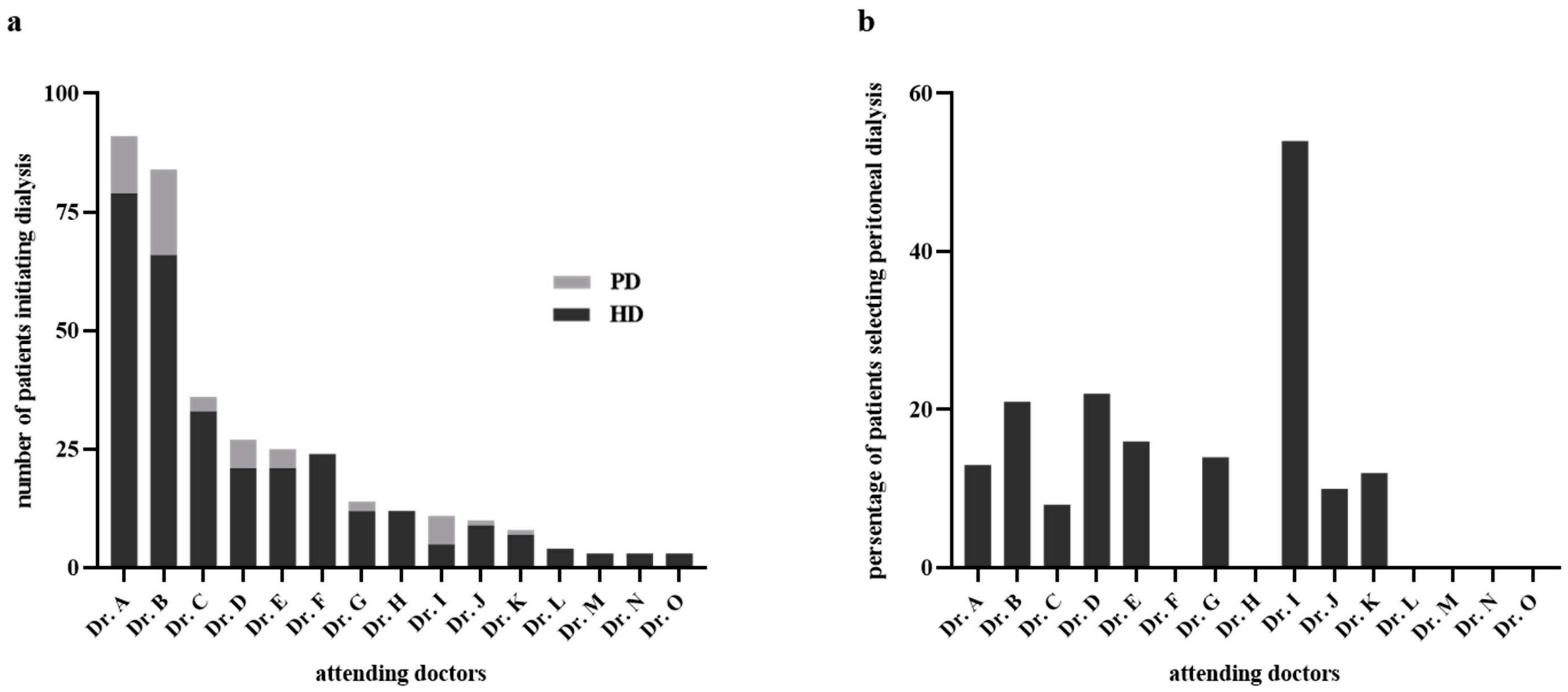

Figure S1. Dialysis modality selection for each attending doctor. Abbreviations: HD, hemodialysis; PD, peritoneal dialysis.

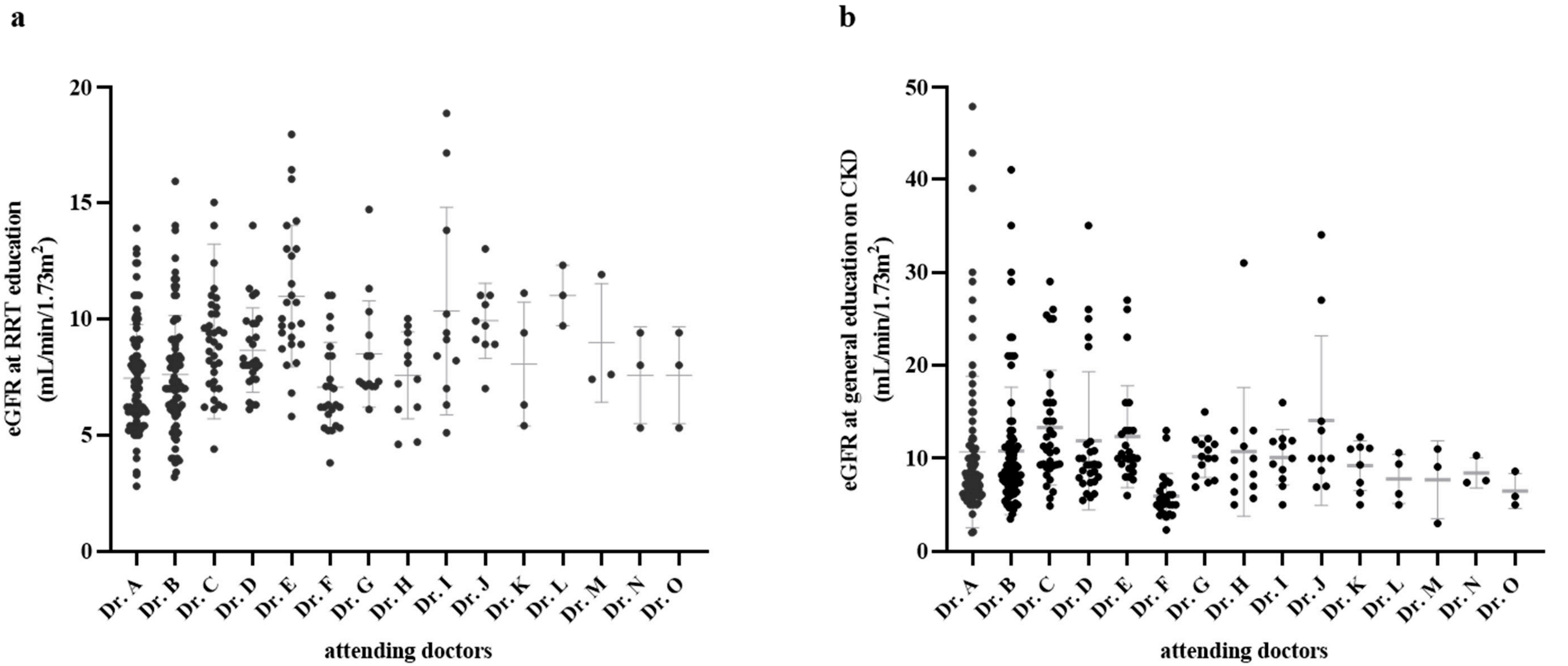

Figure S2. Timing of patient education for each attending doctor. Abbreviations: eGFR, estimated glomerular filtration rate: RRT, renal replacement therapy; CKD, chronic kidney disease. Error bars show mean ± SD.
